# Human Tyrosinase: Temperature-Dependent Kinetics of Oxidase Activity

**DOI:** 10.3390/ijms21030895

**Published:** 2020-01-30

**Authors:** Kenneth L. Young, Claudia Kassouf, Monika B. Dolinska, David Eric Anderson, Yuri V. Sergeev

**Affiliations:** 1 National Eye Institute, National Institutes of Health, 31 Center Drive MSC 2510, Bethesda, MD 20892, USA; 2 National Institute of Diabetes and Digestive and Kidney Diseases, National Institutes of Health, 6 Center Dr. MSC2775, Bethesda, MD 20892, USA

**Keywords:** tyrosinase, protein purification, L-DOPA binding, enthalpy-driven association

## Abstract

Human tyrosinase (Tyr) is involved in pigment biosynthesis, where mutations in its corresponding gene *TYR* have been linked to oculocutaneous albinism 1, an autosomal recessive disorder. Although the enzymatic capabilities of Tyr have been well-characterized, the thermodynamic driving forces underlying melanogenesis remain unknown. Here, we analyze protein binding using the diphenol oxidase behavior of Tyr and van ’t Hoff temperature-dependent analysis. Recombinant Tyr was expressed and purified using a combination of affinity and size-exclusion chromatography. Michaelis-Menten constants were measured spectrophotometrically from diphenol oxidase reactions of Tyr, using L-3,4-dihydroxyphenylalanine (L-DOPA) as a substrate, at temperatures: 25, 31, 37, and 43 °C. Under the same conditions, the Tyr structure and the L-DOPA binding activity were simulated using 3 ns molecular dynamics and docking. The thermal Michaelis-Menten kinetics data were subjected to the van ‘t Hoff analysis and fitted with the computational model. The temperature-dependent analysis suggests that the association of L-DOPA with Tyr is a spontaneous enthalpy-driven reaction, which becomes unfavorable at the final step of dopachrome formation.

## 1. Introduction

Pigmentation is the result of a complex process by which the pigmentary biopolymer, melanin, is synthesized and packed into melanosomes of melanocytes. Various types of oculocutaneous albinism (OCA), a series of autosomal recessive disorders, are associated with reduced pigmentation in the skin, eyes, and hair due to malfunctioning proteins involved in melanogenesis, most notably, human tyrosinase where genetic mutations occur in its corresponding gene *TYR*. Oculocutaneous albinism type 1 (OCA1), the most wide-spread albinism, is caused by bi-allelic mutations in the *TYR* gene and occurs in approximately 1:40,000 people worldwide [1]. This type of albinism is divided clinically into two groups: the most severe type, OCA1A, with disrupted tyrosinase activity and melanin synthesis, or the less severe OCA1B, with residual tyrosinase activity in melanin production. Both subtypes of OCA1 result in numerous changes in clinical phenotypes including reduced best-corrected visual acuity, nystagmus, and others [2]. OCA1 was suggested to be an endoplasmic reticulum (ER) retention disease in which misfolded tyrosinase mutants are retained in the ER by cellular quality control [3]. More than 350 mutations were found in the *TYR* gene as noted in the HGMD Professional 2019.2 database (https://portal.biobase-international.com/hgmd/pro/). Many of these alterations change the production of melanin either by full or partial exclusion of tyrosinase activity. 

Human tyrosinase (hTyr) is a type 1 membrane protein with one alpha helix spanning the transmembrane domain of the melanosome. Human tyrosinase is a glycoprotein, which is post-translationally modified by the addition of several N-linked glycans that are required for protein maturation and stability [4]. Moreover, hTyr is a fully active, monomeric glycoenzyme containing seven sites of *N*-glycosylation that help maintain the stability and functionality of the protein. hTyr can catalyze the initial and rate-limiting steps of the hydroxylation of l-tyrosine (monophenol substrate) into l-3,4-dihydroxyphenylalanine (L-DOPA, diphenol substrate) and the oxidation of o-diphenol into L-dopaquinone [5]. The monophenolase and diphenol oxidase activities are linked to the tyrosinase active site, where two copper atoms (CuA and CuB) are coordinated by six histidine residues [6,7]. 

We previously characterized the enzymatic function of a recombinant intra-melanosomal domain of human tyrosinase (Tyr_tr_) and showed that temperature-sensitive OCA1B-related mutant variants are soluble monomeric glycoproteins with enzymatic activities mirroring the in vivo function [1,8]. In addition, the genetic mutations in tyrosinase, which give rise to OCA1 albinism, show a link to protein conformational stability and protein activity. Recently, we demonstrated that the Tyrp1-mediated protection of human tyrosinase enzymatic activity does not involve stable interactions with tyrosinase domains [9]. Although the enzymatic capabilities of human tyrosinase have been well-characterized, the thermodynamic driving forces underlying melanogenesis remain unknown due to the reaction complexity. Furthermore, the role of temperature in human tyrosinase structure and function is still underestimated. For example, in OCA1B some mutant variants show thermal sensitivity at 31 °C [8]. The role of tyrosinase at the heat shock condition (43 °C) is not clear. Therefore, the understanding of the physiological temperature role in tyrosinase stability and enzymatic activity is important. 

Here, we further characterize protein stability and elucidate the thermodynamic parameters behind the diphenol oxidase behavior of Tyr_tr_ using a simple UV measurement of dopachrome formation at four constant temperatures. We demonstrate, for the first time, that the association of L-DOPA with Tyr_tr_ is a spontaneous enthalpy-driven reaction, suggesting that the initial binding is thermodynamically favorable. By understanding the energetics associated with Tyr_tr_ binding, we can further comprehend how the active site functions, which is pivotal for the search of suitable activators and inhibitors of mutant variants. Moreover, examining the thermodynamics and kinetics of mutant Tyr_tr_ remains vital in understanding the molecular mechanism of oculocutaneous albinism. 

## 2. Results

### 2.1. Protein Purification 

**Figure 1:**
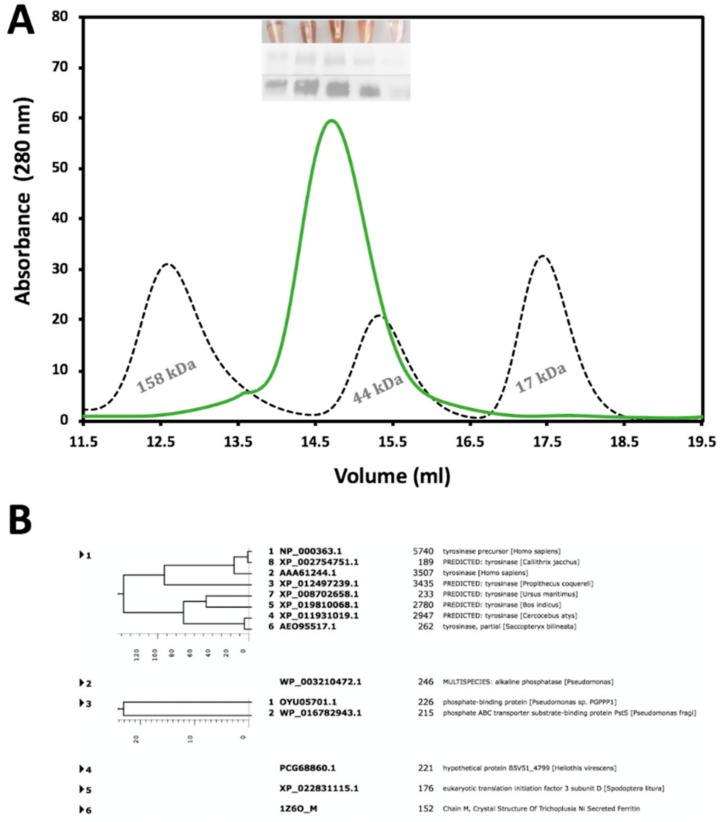
Purification and identification of Tyr_tr_ protein from chromatography and Mass Spectroscopy. Panel (**A**) represents the second purification step using a Superdex 200 Increase 10/300 column with an overly of standards dictating size; gamma-globulin (158 kDa), Ovalbumin (44 kDa), and Myoglobin (17 kDa). Inserts within the figure are L-DOPA colorimetric reactions (Top insert), followed by SDS-PAGE (Middle insert), and Western Blot (Bottom insert). The data refers to the fraction volumes collected for future analysis. In Panel (**B**) the purity of the tyrosinase sample confirmed using mass spectroscopy. In a protein sample, most digested peptides are associated with proteins from the tyrosinase family.

The recombinant Tyr_tr_ was purified by a combination of immobilized metal affinity chromatography (IMAC) and size-exclusion chromatography (SEC) where purification steps were monitored by SDS-PAGE and Western blotting analyses. Moreover, to reduce the time of analysis, fractions eluted from chromatography columns containing active Tyr_tr_ were pinpointed in each step through a colorimetric reaction with L-DOPA. Figure 1 represents the SEC with a Superdex 200 Increase 10/300 column and shows that Tyr_tr_ elutes as a monomeric protein with a molecular weight of approximately 60 kDa. Western blotting of tyrosinase from larval extracts continued to show bands with molecular mass ∼52–64 kDa for Tyr_tr_ and eluted from SEC as a single peak with an approximate molecular mass of ~57 kDa (Figure 1A). The copper color seen in test tubes (Figure 1A, insert Top Panel) indicates fractions with tyrosinase activity corresponding to the singular peak measured at 280 nm, SDS-PAGE (insert Middle Panel), as well as the Western Blot (insert Bottom Panel) obtained from those fractions. An additional purification step using the Superdex 75 10/300 column after 1 h incubation of Tyr_tr_ with 1 M urea was performed before studies and for mass spectrometry analysis. The mass-spectroscopy result has demonstrated a very large number of hits for tyrosinase peptides, significantly above the hits for other proteins, indicating a sample purity as shown in Figure 1B.

### 2.2. ITC Rate Correlates with Dopachrome Activity 

**Figure 2:**
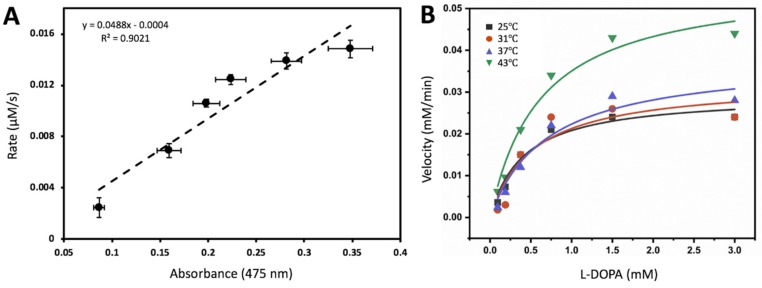
Thermodynamics of tyrosinase association reaction could be recovered from temperature-dependent binding activity. (**A**) Isothermal titration calorimetry rate (µM/s) correlates with the dopachrome absorption. The rate of tyrosinase oxidation of L-DOPA plotted against corresponding L-DOPA concentrations as a function of absorbance values ascertained from Michaelis-Menten plot. (**B**) Diphenol oxidase activity of L-DOPA was measured at different temperatures. Michaelis-Menten constant and V_max_ values for different temperatures is shown in Table 1. Michaelis-Menten equation y = *V_max_* * x/(*K_m_* + x) was used to fit data for each temperature. Temperature-dependent Tyr_tr_ activity (averaged *V_initia_*_l_ and standard errors) used for the evaluation of the Michaelis-Menten constant is shown in Supplementary Material Table S1.

Michaelis-Menten kinetics could be measured using ITC to evaluate simple systems that follow a pseudo first-order rate [10]. From this view, the Michaelis-Menten activities could be implicated to measure apparent thermodynamic parameters of the system. Calorimetric titration of Tyr_tr_ with L-DOPA in a 10 mM sodium phosphate, pH 7.4, is shown in Supplementary Figure S1. The experiment consisted of 20 injections of 12 µL each of a 0.15 mM stock solution of L-DOPA. The oxidation reaction that took place can be visualized by copper color produced from L-DOPA conversion to dopachrome by Tyr_tr_, the insert in Supplementary Figure S1A. As L-DOPA was dropped stepwise into the sample cell, the reaction exhibited an exothermic profile which was converted into a corresponding Michaelis-Menten curve (Supplementary Figure S1B). The enzyme and substrate concentrations were corrected for dilutions when increasing the reaction volume by additional injections. This is exemplified in Supplementary Figure S1C; Michaelis-Menten of L-DOPA in the presence of recombinant Tyr_tr_ that was measured in vitro using UV-spectroscopy at 37 °C, done in tandem with ITC from Figure 2A. The L-DOPA concentrations are exactly ten times higher than six L-DOPA concentration points that can be found in the ITC Michaelis-Menten converted isotherm plot. Figure 2A shows the dopachrome activity measured in absorbance values from these concentration points that were set proportionally to the Rate (µM/s) concentration points ascertained from ITC data where the average ratio value was 20.34 and the Adjusted R^2^ value was 0.90. Thus, the result suggests a correlation between ITC data and dopachrome activity.

### 2.3. Temperature-Dependent Enzymatic Activity

Once the correlation between ITC and UV spectroscopy had been drawn, the enzymatic activities of recombinant Tyr_tr_ were further tested in vitro through UV-spectroscopy at four temperatures: 25, 31, 37, and 43 °C. The parameters of Michaelis-Menten enzymatic reaction, affinity constant *K_m_* and maximal velocity *V_max_* of proteins, were calculated using plots shown in Figure 2B and presented in Table 1 for each temperature analyzed with adjusted R^2^ >0.8.

The Michaelis-Menten curve fitting (Figure 2B) has shown that with L-DOPA as a substrate the diphenol oxidase activity of recombinant Tyr_tr_ displayed both an increasing *K_m_* as well as an increasing *V_max_* as the temperature increased (Table 1). However, the suggested trend does not fit all experimental values. For example, it does not truly apply the mean *K_m_* at 43 °C.

**Figure 3:**
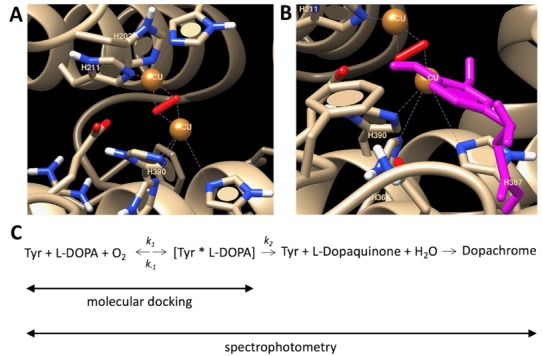
Computational docking of L-DOPA molecule and human tyrosinase. (**A**) Active center of human tyrosinase is shown. (**B**) L-DOPA molecule (magenta) is docked in an active site. (**C**) 4-step reaction of dopachrome formation from L-DOPA, which is catalyzed by Tyr. Symbols *k_1_, k_−1,_* and *k_2_* are catalytic constants. Michaelis constant Km is matching the dissociation constant if assumed that the formation of the product from the tyrosinase-L-DOPA complex occurs at a much slower rate in comparison to the rate of dissociation of the tyrosinase-L-DOPA complex, (i.e., *k_2_* << *k_−1_* of the reaction). Michaelis-Menten kinetics is measured at the dopachrome absorption wavelength (~475 nm). Computational docking measures a binding of L-DOPA in a complex [Tyr*L-DOPA]. Copper atoms and dioxide molecules are shown by orange balls and red rods, respectively.

**Figure 4:**
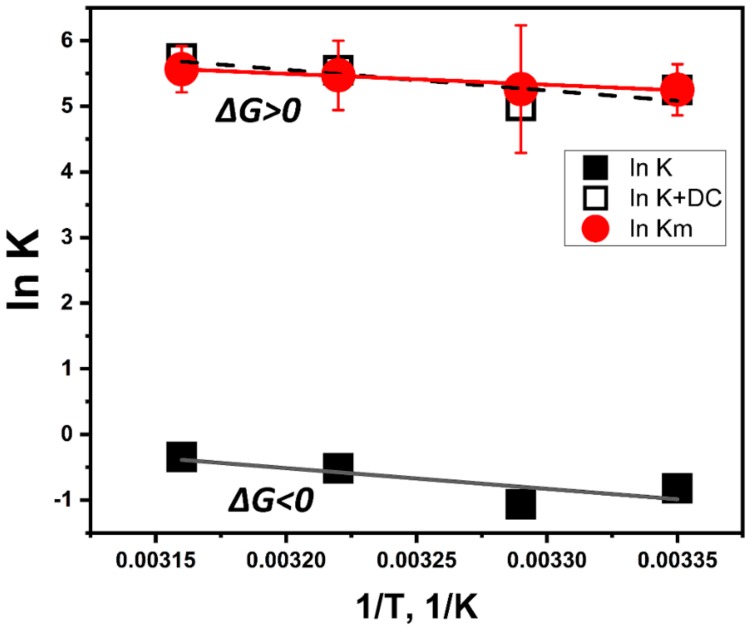
Fitting of temperature-dependent kinetics and computational association data presented in a form of van ‘t Hoff graphs. Michaelis-Menten data shown by red solid circles with error bars and a red trendline. L-DOPA/tyrosinase association was modeled at different temperatures using molecular dynamics and docking simulations. The computational result is shown by black solid squares matched with a black solid line. The model of L-DOPA association was shifted to fit the kinetics data (red solid circles and dashes). The fit is demonstrated by the black open squares and dashes according to the equation: ln *K_m_*(1/T) = ln *K*(1/T) + DC, where DC is the dopachrome effect (DC = 6.07).

To explain the temperature properties of interaction, we performed computer simulations of temperature-dependent association of L-DOPA and the atomic model of Tyr. L-DOPA molecules were docked to the Tyr active site at different temperature conditions. One of the computational experiments is shown in Figure 3 demonstrating a tight binding of L-DOPA and in close vicinity of CuA and CuB atoms in the Tyr active site. Such experiments were repeated at different temperatures. The best L-DOPA docking poses were selected for each temperature and their binding energies are shown in Table 1. A slight rise of the L-DOPA binding seems to confirm a trend found in Michaelis-Menten kinetics. Computer simulations of L-DOPA association suggested a change that could be expected from the van ‘t Hoff analysis of temperature-dependent binding. Indeed, the computational docking model describes the association at the first stage chemical reaction (Figure 3C), when the complex of L-DOPA and Tyr [L-DOPA* TYR] is formed. The association indicates a negative trendline on the van ‘t Hoff plot, shown as a black solid line in Figure 4. This trendline was shifted by the DC value to fit into the Michaelis-Menten kinetics presented by the red solid circles. From the trendlines, apparent thermodynamics parameters of L- DOPA/Tyr association were derived using van ‘t-Hoff relationships [11] as presented in Table 2. Both methods, in silico docking and Michaelis-Menten kinetics, demonstrated similar results: negative enthalpy *∆H* ~21.26–25.72 kJ mol^−1^ and positive entropy *∆S*~0.058−0.078 kJ K^−1^ mol^−1^. This suggests that in silico docking, which is a successful model of the first step of L-DOPA reaction, correlates reasonably well with dopachrome changes measured spectrophotometrically at the last step of reaction (Figure 3C). From our calculations, we could assume that the reaction is spontaneous and is enthalpy-driven [11,12]. Additionally, this reaction could be associated with a partial immobilization of dopachrome.

According to Figure 4, the effect of dopachrome at the last step of the L-DOPA/Tyr interaction shown in Figure 3C could be found as a difference *∆G_dc_ = ∆G_m_ − ∆G*. At room temperature, this value is ~15.05 kJ/mol. This additional free energy works as a switch between two thermodynamic processes. Therefore, the thermodynamically favorable process of L-DOPA association at the first step of the reaction (*∆G* < 0) shows a trend to become thermodynamically unfavorable (*∆G* > 0) at the final step of dopachrome formation. 

## 3. Discussion

Although the enzymatic capabilities of Tyr have been well-characterized, the thermodynamic driving forces underlying melanogenesis remain unknown. Here, we analyze protein binding using the diphenol oxidase behavior of Tyr_tr_ and van ‘t Hoff temperature-dependent analysis. Michaelis-Menten constants were measured spectrophotometrically from diphenol oxidase reactions of L-DOPA at temperatures: 25, 31, 37, and 43 °C. Under the same conditions, the activity and structure of Tyr_tr_ were modeled using 3 ns molecular dynamics and docking. Data were subjected to a van ‘t Hoff analysis and shown to fit with the computational model. We further characterized the stability of Tyr_tr_ and elucidated the thermodynamic parameters behind the diphenol oxidase behavior of Tyr_tr_ using a spectrophotometric measurement. Furthermore, UV spectroscopy showed the last step of dopachrome formation binding is thermodynamically unfavorable (*∆G* > 0); thus, in silico docking may be useful in elucidating binding mechanisms of tyrosinases. 

Both methods, in silico docking and Michaelis-Menten kinetics, presented in Table 2, show similar results with negative apparent enthalpy *∆H* ~21.26–25.72 kJ mol^−1^ and positive apparent entropy *∆S* ~0.058–0.078 kJ K^−1^ mol^−1^. This suggests that in silico docking, which is a successful model of the first step of the L-DOPA reaction reasonably correlates with dopachrome changes measured spectrophotometrically (Figure 3C). A reaction of dopachrome formation is associated with the interaction of L-DOPA with Tyr_tr,_ followed by the release of dopaquinone from the enzyme-substrate complex and, lastly, by the dopachrome and melanin formation. The exact balance of this reaction is difficult to evaluate. However, negative enthalpy, *∆H* < 0, suggests the loss of ionic interactions and hydrogen bonds. Positive entropy, *∆S* > 0, clearly indicates the increase of disorder in the system. This increase could be related to dehydration of interacting molecules, loss of rotational and translational freedom (entropy), or change in their conformation due to the interaction [11,12]. This process is known as the enthalpy-driven reaction (*∆H > T∆S*) [11]. 

Formation of dopachrome, and possibly melanin, is related to the second process. Dopachrome complicates the situation, converting thermodynamically favorable association/dissociation reaction as shown in Figure 4 to the unfavorable process (*∆G* > 0), possibly related to the partial solvation of dopachrome, partial immobilization of dopachrome to itself (melanin formation), and Tyr_tr_. This change might be relatively temperature independent and is most related to the change of enthalpy reaction. Finally, we have shown, for the first time, that the association of L-DOPA and Tyr_tr_ is spontaneous, enthalpy-driven, and thermodynamically favorable.

Presented in this work, the spectrophotometric van ‘t Hoff technique provides a simplified way to measure the apparent thermodynamic signature of a protein sample. Thermal parameters of protein interactions could be determined by measurement of association constants at different temperatures followed by the analysis using the van ‘t Hoff equation. Here, we assume that the formation of the product from the tyrosinase-L-DOPA complex occurs at a much slower rate in comparison to the rate of dissociation of the tyrosinase-L-DOPA complex. In this condition, values of affinity and dissociation constants are equal. Therefore, the van ‘t Hoff equation could be used to determine a thermodynamic signature of L-Dopa binding to the tyrosinase active site. The thermodynamic signature of protein could be measured using ITC. ITC is a technique that can directly measure the binding energetics of biological processes like protein-ligand binding where Gibbs energy, enthalpy, and entropy associated with binding can be precisely determined [13]. Many proteins studied are targets for pharmaceutical drug development, underscoring the prominent role of ITC in drug design. However, the ITC technique seems to be less reliable for the thermodynamic analysis of tyrosinase reaction (Figure 3C). Indeed, the tyrosinase reaction is a complex process, which creates a dopachrome, a pigment-like substance with high heat absorption. The overall reaction of melanin formation and disruption of the tyrosinase hydration shell likely overshadow the individual binding kinetics of L-DOPA. This limitation does not allow precise measurement of L-DOPA binding to tyrosinase during the ITC run at different temperature conditions. However, this difficulty could be avoided if a temperature-dependent measurement could be performed spectrophotometrically as demonstrated in this work. Moreover, we showed that from these experiments all major parameters of L-DOPA binding including the reaction thermodynamics signature could be successfully recovered from the van ‘t Hoff analysis. 

In the future, we plan to apply thermal analysis for the quantitative comparison of functional activity of albinism-related mutant variants, express-characterization of tyrosinase substrates and inhibitors using a combination of computational methods, and activity measurements performed using the regular plate-reader. This approach could be essential in the understanding of tyrosinase functions and the search for a cure for inherited diseases using this protein as a target in drug screens and future structural studies. 

In conclusion, we believe that the measurement of temperature-dependent activities will be an easier way to understand the thermodynamics of complex chemical reactions of melanin formation. By understanding the energetics associated with Tyr_tr_ binding, we can further comprehend how the active site functions, which is pivotal for the search of suitable activators and inhibitors of mutant variants. Lastly, examining the thermodynamics and kinetics of mutant Tyr remains vital in understanding the molecular mechanism of oculocutaneous albinism.

## 4. Materials and Methods 

### 4.1. Expression, Isolation, and Identification of Tyrosinase 

Purification of human recombinant tyrosinases was described previously [8,14,15]. Briefly, the Tyr_tr_ (residues 19–469 of the native protein) were expressed in baculovirus and produced in whole insect *Trichoplusia. ni (T. ni)* larvae. Tyr_tr_ was isolated using a 6xHis-tag with few modifications. Frozen at −80 °C, infected larvae were homogenized in 5 × (vol/weight) lysate buffer (20 mM sodium phosphate, pH 7.4, 500 mM NaCl, 5 mM imidazole, 25 µM 1-Phenyl-2-thiourea, PTU (Sigma-Aldrich, Saint Louis, MO, USA), 2 mM MgCl_2_, 40 µg/mL DNAse I (Thermo Fisher Scientific, Waltham, MA, USA), 0.2 mg/mL lysozyme. Complete set of protease inhibitors was obtained (Roche, San Francisco, CA, USA). After 15 min of incubation on a rotator, lysates were sonicated for 10 min and centrifuged at 8000 RPM for 30 min at 4 °C. Supernatants were then diluted 1:1 (*v*/*v*) with binding buffer (20 mM sodium phosphate, pH 7.4, 500 mM NaCl, 5 mM imidazole) and purified by immobilized metal affinity chromatography (IMAC) followed by size-exclusion chromatography (SEC) using ÄKTA Pure Protein Purification System (GE Healthcare, Silver Spring, MD, USA). 

Lysate was loaded on a 5 mL His-Trap FF Crude IMAC column (GE Healthcare) equilibrated with binding buffer and eluted with an instant 500 mM imidazole buffer switch for elution. Fractions containing the protein of interest were dialyzed overnight against 4 L of SEC buffer (50 mM Tris-HCl, pH 7.4, 1 mM ethylenediaminetetraacetic acid (EDTA), 150 mM NaCl, 50 µM Tris(2-carboxyethyl)phosphine hydrochloride (TCEP) and concentrated using Amicon Ultra 10,000 MWCO centrifugal filter units (Merc Millipore, Burlington, MA, USA). Proteins were further purified by SEC using Sephacryl S-200 HR 16/60 and Superdex 200 Increase GL 10/300 columns (GE Healthcare). 

The columns were calibrated with SEC standards (Bio-Rad, Hercules, CA, USA): thyroglobulin, γ-globulin, ovalbumin, myoglobin, and vitamin B12. The location of Tyr_tr_ in the various column fractions was monitored by SDS-PAGE using 4–15% polyacrylamide gels (Bio-Rad). Protein identity and purity were confirmed by Western blot analysis using anti-tyrosinase (T311) antibodies (Santa Cruz Biotechnology, Dallas, TX, USA) as well as by mass spectrophotometry. Tyr_tr_ concentration after each step of purification was determined at A_280nm/260nm_ using the NanoDrop 2000c UV-Vis spectrophotometer (Thermo Scientific, Waltham, MA, USA).

### 4.2. Tyrosinase Diphenol Oxidase Activity

Dopachrome formation was created by the oxidase activity of Tyr_tr_ and measured spectrophotometrically at 475 nm using the SpectraMax i3 multi-mode detection platform (Molecular Devices, San Jose, CA, USA) [16,17]. Tyr_tr_ was incubated in the presence of 1.5 mM L-DOPA (Sigma-Aldrich) in 10 mM Sodium Phosphate (pH 7.4) at each of the following temperatures: 25, 31, 37, and 43 °C. The temperatures within the microplate chamber were solely monitored and maintained by the SpectraMax i3 device and the corresponding software. The device has a temperature accuracy at ±1 °C and temperature uniformity across the entire plate ±0.75 °C. The buffer was pre-warmed to corresponding temperatures via water bath before plate placement. Additionally, the SpectraMax device was also pre-equilibrated before experiments were carried out in a microplate chamber. Measurements in duplicates at each of four temperatures were independently repeated three times. Optical densities from duplicate reactions at the temperatures were averaged after subtraction of baseline activity. 

### 4.3. Michaelis-Menten Kinetics 

To process the activity data, absorbance measurements (mOD) of dopachrome formation were converted to concentration measurements (mM) using Beer-Lambert law with ε dopachrome = 3700 M^−1^ cm^−1^ and L = 0.3 cm. For Michaelis-Menten kinetics, absorbance measurements were performed at the following L-DOPA concentrations: 0.9375, 0.1875, 0.375, 0.75, 1.50, 3, and 6 mM (with in-tandem ITC UV spectroscopy being an exception where L-DOPA concentrations were: 10.9, 21.9, 43.8, 87.5, 175, 350 µM). 

The first eight minutes of every reaction was used to find the initial velocity, *V_initial_*. Then, using Michaelis-Menten nonlinear polynomial fit on OriginPRO Software (version 9.0, OriginLAB Corporation, Boston, MA, USA), the association of the reaction known as a Michaelis-Menten constant, *K_m_*, and the maximum rate of reaction, *V_max_*, were calculated. This calculation was repeated for each reaction to produce three data sets for each temperature condition. Finally, *K_m_* and *V_max_* were averaged over duplicate reactions at the same temperature, providing a total of 12 *K_m_* and *V_max_* values for each data set.

### 4.4. In-Silico L-DOPA Binding 

The tyrosinase model, Tyr.pdb, was downloaded from the ocular proteomics web site (https://neicommons.nei.nih.gov/#/proteome). The model was used to better elucidate the mechanism of human tyrosinase and L-DOPA binding. The Tyr was subjected to 3 nanoseconds of molecular dynamics (MD) in YASARA (www.yasara.org), with conditions pH = 7.2, 0.9% (155 mM) NaCl, and a variable water density such that the pressure of the environment remained at 1 bar at the four different temperatures: 25, 31, 37, and 43 °C. The above conditions were used to stabilize the protein atomic structure during the MD simulations at a certain temperature. After MD, the different PDB files were subjected to YASARA’s molecular docking script dock_run.mcr. The YASARA script was modified so that 200 docking runs were analyzed for each receptor-ligand pair instead of the standard 25. The script uses AutoDock Vina, a gradient-optimization method holding the receptor (i.e., tyrosinase) rigid and the ligand (i.e., L-DOPA) flexible. It uses a statistical scoring function to give each the binding conformation (of receptor and ligand) a binding affinity and binding energy without any assumption about pH or 0.9% salt conditions. The binding affinities were used to determine association constants at four different temperatures and to create a van ‘t Hoff plot. The differences in binding energy due to pHs (pH7.2 and pH7.4) were in limits of statistical error. However, in experimental conditions, 0.9% NaCl could partially inhibit the catalytic activity of human tyrosinase [8] As a result, the Vmax value will be lower (~40%), but the affinity of binding and the respective *K_m_* constant might change in significantly less degree. Therefore, we expect that this difference does not influence the major conclusion of the analysis.

### 4.5. van ‘t Hoff Analysis

The thermodynamic signature of L-DOPA binding was determined by measurement of affinity constants at different temperatures followed by an analysis using the van ‘t Hoff equation. To perform the van ‘t Hoff analysis, temperature-dependent data obtained for Michaelis-Menten kinetics and in silico binding were graphed on a van ‘t Hoff plot. A straight line was then fit through the four temperature points using OriginPRO Software (version 9.0, OriginLAB Corporation). The line could be interpreted with the following equation:

*ln (K/[E]) = (−∆H/R) 1/T + ∆S/R* (1)

where temperature *T* is measured in Kelvin (K), *R* is a gas constant (*R* = 8.31 J/K/mol), and *−∆H/R* and *∆S/R* are the slope and intercept of the line, respectively. Here, *K* is an association constant (reciprocal to the dissociation constant) normalized by tyrosinase concentration, *[E].*

### 4.6. Isothermal Titration Calorimetry (ITC) 

The ITC experiments were performed in 10 mM Sodium Phosphate buffer, pH 7.4 at 37 °C. For the experiments, 0.15 mM L-DOPA was placed into the syringe of the Nano ITC 2G (TA Instruments, New Castle, DE, USA). Using the Multiple Injection Method (MIM) format, a small volume (12 µL) of the L-DOPA solution was injected into the sample cell (volume = 1.5 mL) containing 0.1 mg/mL of Tyr_tr_ at 37 °C. The injections were made over a period of 10 s with a 200-s interval between subsequent injections. The samples were stirred at 350 rpm, degassed, and placed in the reference cell. The provided titration curve was converted into Michaelis-Menten format using TA Nano Analyze Software (version 1.2.0, TA Instruments). Finally, UV-spectroscopy Michaelis-Menten at 37 °C was done tandemly and correlated to ITC data. For these Michaelis-Menten kinetics, absorbance measurements were performed at the following L-DOPA concentrations: 0.9375, 0.1875, 0.375, 0.75, 1.50, 3, and 6 mM. The absorbance values from these six concentration points were set proportionally to the rate (µM/s) concentration points ascertained from the ITC data where the average ratio value was 20.34 and the Adjusted R^2^ value was 0.90. 

### 4.7. Mass-Spectroscopy

Following a Superdex 200 Increase GL 10/300 column (GE Healthcare) run, Tyr_tr_ was incubated at room temperature for 1 h in the presence of 1 M Urea and was placed on the Superdex 75 10/300 column. Concentrated fractions of Tyr_tr_ were run on SDS-PAGE using 4–15% polyacrylamide gels (Bio-Rad). The gel was stained using the Novex Colloidal Blue Stain Kit (Invitrogen, Waltham, MA, USA). Liquid and in-gel digestions were performed on pre-alkylated protein bands using a method previously described [18] except for using acid extractable Sodium Dodecanoate [19]. Samples were off-line purified using Stage Tips [20]. Data collected by LC/MS/MS were analyzed using Mascot [21] against the extant NCBI protein database without species specificity.

## Figures and Tables

**Table 1:** Parameters of Michaelis-Menten kinetics and results of computational binding.

Temperature (℃)	Michaelis-Menten Kinetics			Energy Constant	Computational Docking		
	*K_m_* (mM)	*V_max_* (mM/min)	Adj. R^2^	*ΔG_d_* (kJ/mol)	Correctly Docked L-DOPA	Binding Energy (kJ/mol)	Binding Affinity, *K_d_* (mM)
25	0.41 ± 0.11	0.029 ± 0.003	0.95	−11.57 ± 2.97	005	25.71	0.032
31	0.52 ± 0.31	0.032 ± 0.007	0.83	−12.74 ± 2.90	002	26.75	0.011
37	0.70 ± 0.25	0.038 ± 0.005	0.94	−13.32 ± 2.87	002 009	26.71 25.21	0.019
43	0.62 ± 0.15	0.057 ± 0.003	0.96	−13.90 ± 2.83	003	26.00	0.016

Note: Correctly docked L-DOPA assumes the pose of L-DOPA molecule located in the tyrosinase active site.

**Table 2:** Results of evaluation of apparent enthalpy and entropy of tyrosinase and L-DOPA association obtained from van ‘t Hoff model.

Parameters	Michaelis-Menten Kinetics	L-DOPA Docking
**Apparent Enthalpy Change, *ΔH*,** **kJ mol^−1^**	−21.26 ± 6.18	−25.72 ± 13.96
**Apparent Entropy Change, *ΔS*,** **kJ K^−1^ mol^−1^**	0.058 ± 0.020	0.078 ± 0.045
**Pearson’s r**	−0.91	−0.79
**Adjusted R^2^**	0.75	0.44

## Supplementary Materials

Supplementary materials can be found at https://www.mdpi.com/1422-0067/21/3/895/s1.

## Abbreviations

SEC	Size Exclusion Chromatography
IMAC	Immobilized Metal Affinity Chromatography
ITC	Isothermal Titration Colorimetry
L-DOPA	L- 3, 4-Dihydroxyphenylalanine
hTyr	Human Tyrosinase
Tyr_tr_	Human Tyrosinase Intra-Melanosomal Domain
OCA1	Oculocutaneous Albinism 1
