# Supplementary material for: Human Tyrosinase: Temperature-Dependent Kinetics of Oxidase Activity
Source: Int J Mol Sci. 2020 Jan 30;21(3):895. doi: 10.3390/ijms21030895 (PMC7037427; doi:10.3390/ijms21030895)
Supplement: Supplementary file 1 [file ijms-21-00895-s001.pdf]

Supplementary Materials

Supplementary Table S1. Temperature dependent $hTyr_{tr}$ activity ($V_{initial}$) used for the evaluation of the Michaelis-Menten constant.

**[L-DOPA], mM**	**$V_{initial,}$ mM/min**			
	**25°C**	**31°C**	**37°C**	**43°C**
**0.09375**	0.0036 ± 0.0017	0.00180 ± 0.00065	0.0024 ± 0.00065	0.0062 ±0.0028
**0.1875**	0.0073 ± 0.0037	0.0030 ± 0.0012	0.0060 ± 0.0014	0.0096 ± 0.0036
**0.375**	0.015 ± 0.0082	0.015 ± 0.0072	0.012 ± 0.0053	0.021 ± 0.0097
**0.75**	0.021 ± 0.012	0.024 ± 0.013	0.022 ± 0.011	0.034 ± 0.017
**1.5**	0.024 ± 0.014	0.026 ± 0.014	0.029 ± 0.015	0.043 ± 0.022
**3.0**	0.024 ± 0.015	0.024 ± 0.012	0.028 ± 0.014	0.044 ± 0.021
**6.0**	0.023 ± 0.013	0.024 ± 0.011	0.026 ± 0.012	0.041 ± 0.019

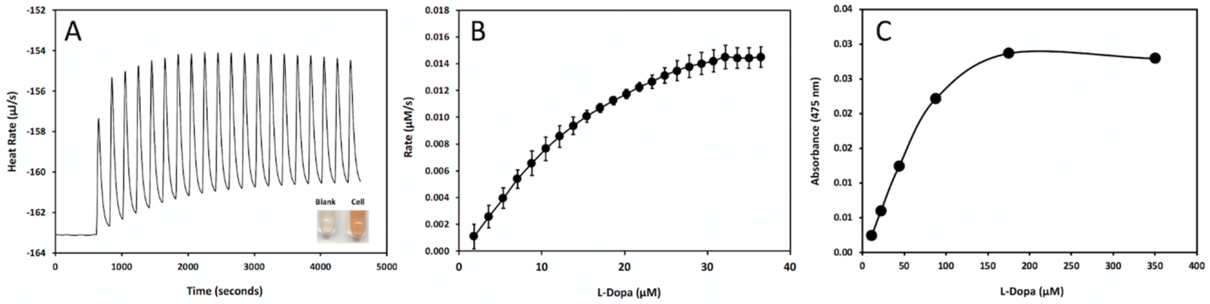

Figure S1. Isothermal Titration Calorimetry Conversion to Michaelis-Menten.

Panel A: Differences between the sample and reference cell containing deionized water. The heat dilution of L-DOPA into 10 mM sodium phosphate buffer has been subtracted. Insert shows tyrosinase oxidation of L-DOPA compared to buffer blank measured at 37°C. **Panel B**: The data from **Panel A** has been transformed into reaction rate (µM/s) and plotted against their corresponding L-DOPA concentrations through TA Nano Analyze Software. **Panel C**: UV spectroscopy derived Michaelis-Menten plot of diphenol-oxidase activity of Tyr_{tr} as a function of L-DOPA concentrations. Assay was measured at 37°C using 475 nm for the formation of dopachrome as a metric for enzyme activity.
